# Electrical impedance myography for the early detection of muscle ischemia secondary to compartment syndrome: a study in a rat model

**DOI:** 10.1038/s41598-023-45209-w

**Published:** 2023-10-25

**Authors:** Aron Lechtig, Philip Hanna, Janice A. Nagy, John Wixted, Ara Nazarian, Seward B. Rutkove

**Affiliations:** 1 Musculoskeletal Translational Innovation Initiative, Carl J. Shapiro Department of Orthopaedic Surgery, Beth Israel Deaconess Medical Center, Harvard Medical School, Boston, MA USA; 2 Department of Neurology, Beth Israel Deaconess Medical Center, Harvard Medical School, 330 Brookline Avenue, Boston, MA 02215 USA; 3 Department of Orthopaedic Surgery, Yerevan State Medical University, Yerevan, Armenia

**Keywords:** Biomedical engineering, Experimental models of disease, Musculoskeletal system, Skeletal muscle

## Abstract

Acute Compartment Syndrome (ACS) is one of the most devastating orthopedic conditions, affecting any of the body’s many compartments, which, if sufficiently severe, may result in disability and amputation. Currently, intra-compartmental pressure measurements serve as the gold standard for diagnosing ACS. Diagnosing limbs at risk for ACS before irreversible damage to muscle and nerve is critical. Standard approaches for diagnosing impending compartment syndrome include clinical evaluation of the limb, such as assessment for “tightness” of the overlying skin, reduced pulses distally, and degree of pain, none of which are specific or sensitive. We have proposed a novel method to detect ACS via electrical impedance myography (EIM), where a weak, high-frequency alternating current is passed between one pair of electrodes through a region of tissue, and the resulting surface voltages are measured via a second pair. We evaluated the ability of EIM to detect early muscle ischemia in an established murine model of compression-induced muscle injury, where we collected resistance, reactance, and their dimensionless product, defined as Relative Injury Index (RII) during the study. Our model generated reproducible hypoxia, confirmed by Hypoxyprobe™ staining of endothelial regions within the muscle. Under conditions of ischemia, we demonstrated a reproducible, stable, and significant escalation in resistance, reactance, and RII values, compared to uninjured control limbs. These data make a reasonable argument for additional investigations into using EIM for the early recognition of muscle hypoperfusion and ischemia. However, these findings must be considered preliminary steps, requiring further pre-clinical and clinical validation.

## Introduction

Acute Compartment Syndrome (ACS) remains one of the most devastating of orthopedic conditions^1^. With an average incidence of approximately 7.3/100,000 males and 0.7/100,000 females, full-blown compartment syndrome can result in disability, which, if sufficiently severe, may warrant amputation^2,3^. The condition can affect any of the body’s many compartments—regions with fixed volumes, anatomically separated from other areas by inflexible fascial planes. The most common location for acute compartment syndrome is the anterior tibial compartment, usually resulting from a tibial fracture or crush injury, though several other regions can also be affected, including but not limited to compartments of the gluteal region, posterior tibial, thigh, forearm, hand and foot^1,3,4^. Pressure builds in the compartment due to swelling from an injury, eventually exceeding capillary fill pressure. This causes impaired perfusion and ischemia, ultimately resulting in necrosis of multiple tissues, including muscle and nerve^5^.

Diagnosing limbs at risk for compartment syndrome before irreversible damage to muscle and nerve is critical. Standard approaches for diagnosing impending compartment syndrome require clinical evaluation of the limb, including assessment for “tightness” of the overlying skin, reduced pulses distally, and the degree of pain^3,6^. Currently, intra-compartmental pressure measurements serve as the gold standard for diagnosing ACS. However, such clinical assessment approaches are fairly non-specific and insensitive; compartment syndrome remains a clinical diagnosis based on pain, swelling, and the clinical judgment of individual providers. One of the few agreed-upon objective measurements of compartment syndrome requires the placement of a needle-pressure gauge to assess the intracompartment pressure, and the current criteria for compartment syndrome is an absolute value of intra-compartmental pressures above 30 mm Hg, or less than 30 mm Hg differential between the diastolic blood pressure of the patient and the intra-compartmental pressure^3^. Interestingly, this measures pressure, not tissue perfusion, and only indirectly provides information about tissue viability within the affected compartment. Difficulty obtaining and interpreting invasive compartment pressure measurements has limited the clinical usefulness of these measurements, where they are only commonly used in situations where obtunded patients cannot provide an accurate clinical picture of evolving compartment syndrome and typically remain a single point-in-time measurement. While clinical judgment remains the gold standard for diagnosing ACS, inexperienced providers or those unfamiliar with ACS may not even consider the diagnosis of the condition until after ischemia and irreversible damage have already occurred.

Indeed, many patients with leg injuries insidiously develop compartment syndrome, which might be easily obscured by opioid and other analgesic therapies^7–9^. Thus, a portable, wireless, non-invasive approach for monitoring the development of compartment syndrome would have substantial clinical value.

Other approaches have also been suggested, including those based on alterations in ultrasound echo intensity, shear wave elastography, and regional tissue oxygen saturation via near-infrared spectroscopy (NIRS)^6^. However, most of these approaches suffer from substantial limitations in that they need to be applied in the emergency room by physicians, and they cannot be readily used to assess patients over time^10^. Conversely, NIRS offers an alternative where sensors can continuously monitor limb tissue oxygenation levels in real time.

One approach that has not been studied is the application of electrical impedance myography (EIM). In EIM, a weak, high frequency, directionally-specific, alternating current is passed between one pair of electrodes (the current emitting electrodes) through a region of tissue, and the resulting surface voltages over that region are measured via a second pair (the voltage measuring electrodes) from which the impedance characteristics, including the resistance, reactance, and phase angle, are measured. The technology has already shown substantial value in several neuromuscular disorders, including amyotrophic lateral sclerosis and muscular dystrophy^11–15^. It has also been shown to be sensitive to acute injury, including muscle stretch^16^. Additionally, other forms of electrical bioimpedance have been shown to be highly sensitive to cardiac ischemia^17,18^. Thus, it logically follows that EIM methods may be very sensitive to the development of compartment syndrome—another form of ischemic injury—this time impacting skeletal rather than cardiac muscle.

Therefore, here we aim to evaluate the ability of EIM to detect early muscle ischemia in an established murine model of compression-induced muscle injury^19,20^. Understanding that acute compartment syndrome is not a pure pressure problem but a hypoxic and ischemic phenomenon, we *hypothesize that EIM measurements are sufficiently sensitive to detect early changes in compression-induced murine muscle ischemia.*

## Materials and methods

### Surgical procedure to introduce ischemic injury

The project was approved by the Institutional Animal Care and Use Committee (IACUC) at Beth Israel Deaconess Medical Center (BIDMC) following all regulatory (Animal Research: Reporting of In Vivo Experiments (ARRIVE)) and institutional guidelines. Twenty male, adult (13-weeks-old) Sprague Dawley rats were used in this study (Charles River Laboratories, Boston, MA, USA). Fifteen animals were subjected to ischemic injury, and the other five animals were used as non-injury controls. All animals were housed in the animal facility at BIDMC and provided food and water ad libitum. The animals were allowed a minimum of 72 h to acclimatize before receiving any intervention.

**Figure 1:**
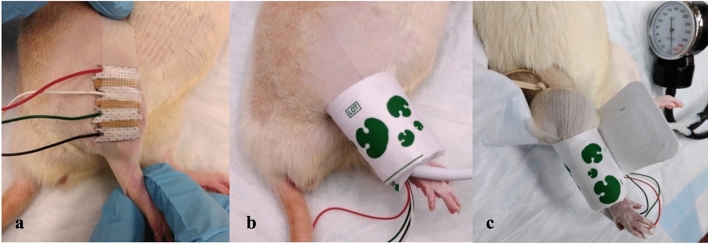
Experimental set up for ischemia induction and EIM measurement in the right hind limb. After the skin is shaved, electrodes are attached to the skin over the anterolateral muscle compartment (**a**). Size 3 neonatal blood pressure cuff is placed around the leg (**b**). Elastic rubber band is tightened around the upper thigh and the blood pressure cuff is inflated to 300 mmHg (**c**).

The animals were anesthetized using isoflurane at 5% in an induction chamber, then maintained at 2% via a nose cone. After induction, rats were placed on a circulating water heating pad (Gaymar T/P 500, Stryker, Kalamazoo, MI, USA) in the left lateral decubitus position. They were then prepared by shaving the right hindlimb using a coarse- followed by fine-hair clippers. Extreme care was taken to prevent skin abrasions during the hair removal process. Four Ambu Neuroline 700 solid-gel adhesive surface Ag/AgCl electrodes (70010-K/C/12; Ambu, Inc. Columbia, MD, USA) were cut into 2-mm wide sections using a razor blade. Spaced 2 mm apart, the non-adhesive surfaces of the electrodes were attached to a 1 × 10 cm strip of adhesive tape. Rubber sections were attached to the adhesive tape, filling the 2 mm gaps between the electrodes to act as insulators (Fig. 1a). The anterolateral compartment of the leg was palpated between the knee and the ankle joints. After removing the protective cover, the adhesive gel side of the electrodes was attached to the skin over the anterolateral compartment in a fashion perpendicular to the long axis of the leg. Once the appropriate positioning of the probes was confirmed, a size 3 neonatal blood pressure cuff (Trimline Medical Products, Branchburg, NJ, USA) was placed around the leg (Fig. 1b) and inflated to 300 mm Hg.

Hind limb ischemia was induced in 15 animals as described previously^19,20^ (ischemia group). Briefly, a 5 mm wide × 1 mm thick rubber band was used as a tourniquet to induce arterial ischemia in the right hindlimb. The band was stretched and applied around the upper thigh as closely as possible to the groin and then tightened to maximum and double-knotted. Immediate paleness in the toes of the right hindlimb confirmed the ischemia. The tourniquet and the inflated pressure cuff were maintained for 40 min to ensure acute arterial ischemia (Fig. 1c).

### Electrical impedance myography measurements

EIM measurements were performed with a Myolex mView system (Myolex, Boston, MA, USA), using a previously described animal adaptor^21^. Measurements were collected immediately after inflating the cuff (baseline), immediately after the tourniquet was applied (inflated tourniquet), followed by measurements every two minutes for the first ten minutes, every 5 min for the following 20 min, and finally once at the end of forty minutes of ischemic injury. At the end of the experiment, the rats were immediately exsanguinated via a cardiac puncture through the right chest wall utilizing an 18-gauge needle attached to a 20-cc syringe. The syringe was replaced once it was full, and exsanguination was continued until euthanasia was confirmed visually by the lack of respiratory movement.

For the five non-injury control animals (control group), the same procedures for placement of the EIM electrodes were used without inducing ischemia. EIM measurements were made at a non-injury baseline (control) by inflating the blood pressure cuff to 30 mm Hg to provide consistent contact between the electrodes and the animal skin.

A set of five measurements was collected for each time point described above. Resistance (R, Ω) and reactance (X, Ω) values, the outputs from the device, and calculated from the measured voltage characteristics, were collected over a frequency range of 10 kHz to 1 MHz. Rather than using absolute impedance values, which can vary to some extent depending on the exact interelectrode distances, the size of the animal, and limb position, we chose to compare changes over time relative to baseline. Based on previous cardiac ischemia data, during persistent ischemia, R and X are anticipated to increase over time. To amplify the effect of alterations in these two values, we introduced the Injuy Index (R_t_ × X_t_), and then the dimensionless Relative Injury Index at time* t* (RII_t_):

1 $${RII}_{t}=\frac{{R}_{t}\times {X}_{t}}{{R}_{i}\times {X}_{i}}$$

where R_t_ is single frequency resistance t time t, X_t_ is single frequency reactance at time t, R_i_ is single frequency resistance at baseline, and X_i_ is single frequency reactance at baseline. This simple index helps to amplify the impact of ischemic change.

### Immunohistochemistry

To validate the occurrence of tissue ischemia by our model, a subset of the “ischemic injury” animals (n = 6) received pimonidazole HCl (Hypoxyprobe-1, HPI, Inc., Burlington, MA, USA), injected intraperitoneally at a dose of 100 mg/kg, 90 min before initiation of ischemia. Pimonidazole HCl is distributed to all tissues via systemic circulation and forms irreversible bonds with intracellular and extracellular proteins under hypoxic conditions. At the end of the experiment and before euthanasia, the muscles of the anterolateral and posterior compartments of the ischemic and contralateral sides were harvested. The ischemic side was used as our experimental side, and the contralateral side was used as their own control to ensure the comparison occurred under the same systemic conditions regarding oxygenation and metabolic status. Muscles were immediately fixed in 4% paraformaldehyde. Twenty-micron thick paraffin-embedded sections were processed and stained using HP-Red549-Kit (Hypoxyprobe-1, HPI, Inc., Burlington, MA, USA) per manufacturer’s instructions^22,23^.

### Data analysis

The Shapiro–Wilk test for normality was used to evaluate the data distribution. A repeated measures one-way analysis of variance (ANOVA) with Bonferroni confidence interval adjustment was performed to assess changes in reactance, resistance, and RII over time. Estimated marginal means were used to assess within-subject pairwise comparisons. Student’s T-test was used to assess the difference between the control and ischemia groups. The SPSS software (IBM-SPSS, version 26.0, Armonk, NY, USA) was used for data analysis. All comparisons were two-tailed, and a p-value less than 0.05 was considered statistically significant.

## Results

**Figure 2:**
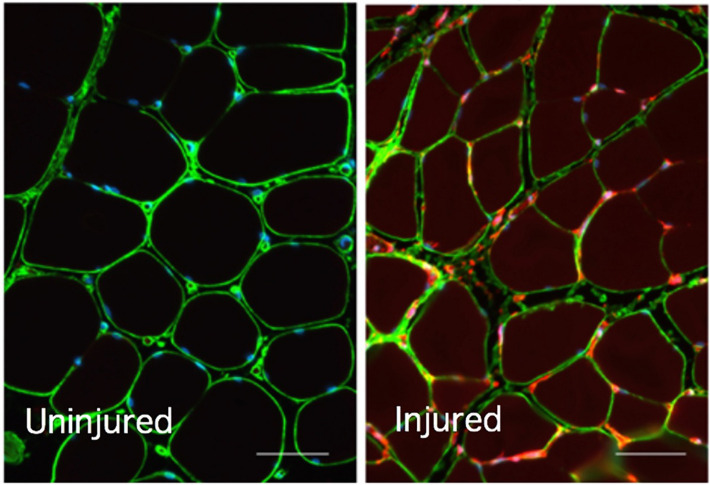
Evidence of ischemic injury; collagen VI staining(green) and Hypoxyprobe™ probe staining (red) in the tibialis anterior muscle of the ACS injured right hind limb (right panel) and the lack of Hypoxyprobe probe staining (red) in the uninjured left hind limb (left panel) from the same animal. Bar = 50 microns.

**Figure 3:**
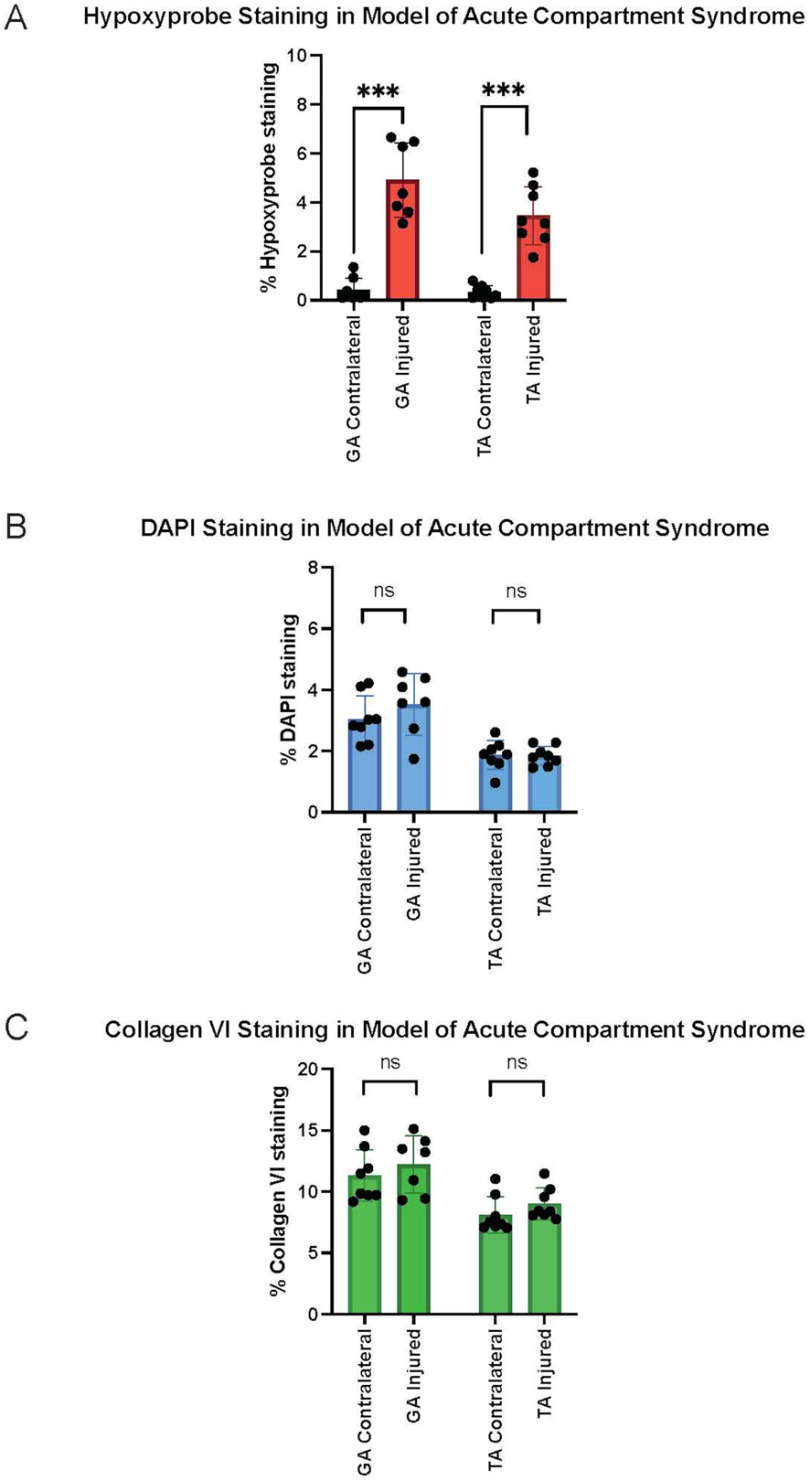
Quantification of hypoxyprobe-1 (**a**), DAPI (**b**), and collagen VI (**c**) staining for tibialis anterior (TA) and gastrocnemius (GA) muscles for injured and contralateral limbs. ***p < 0.001; *ns* not significant.

Hypoxyprobe-1 staining demonstrated evidence of ischemia impacting both the muscle-associated vasculature (Intense red staining) as well as the myocytes (diffuse red staining) in the limb of a representative image from an animal experiencing induced ischemia (Fig. 2, right panel), while the contralateral uninjured limb from the same animal showed no evidence of hypoxia (no red staining) (Fig. 2, left panel). These results support using this model as a reasonable proxy for the early onset of ACS in rats to test the proposed EIM technology. Figure 3 provides quantitative assessments of Hypoxyprobe-1 (a), DAPI (b), and collagen VI (c) staining, where Hypoxyprobe-1 staining was significantly different between the injured and contralateral limbs for both tibialis anterior (TA) and gastrocnemius (GA), but not for DAPI or collagen VI as would be expected.

**Figure 4:**
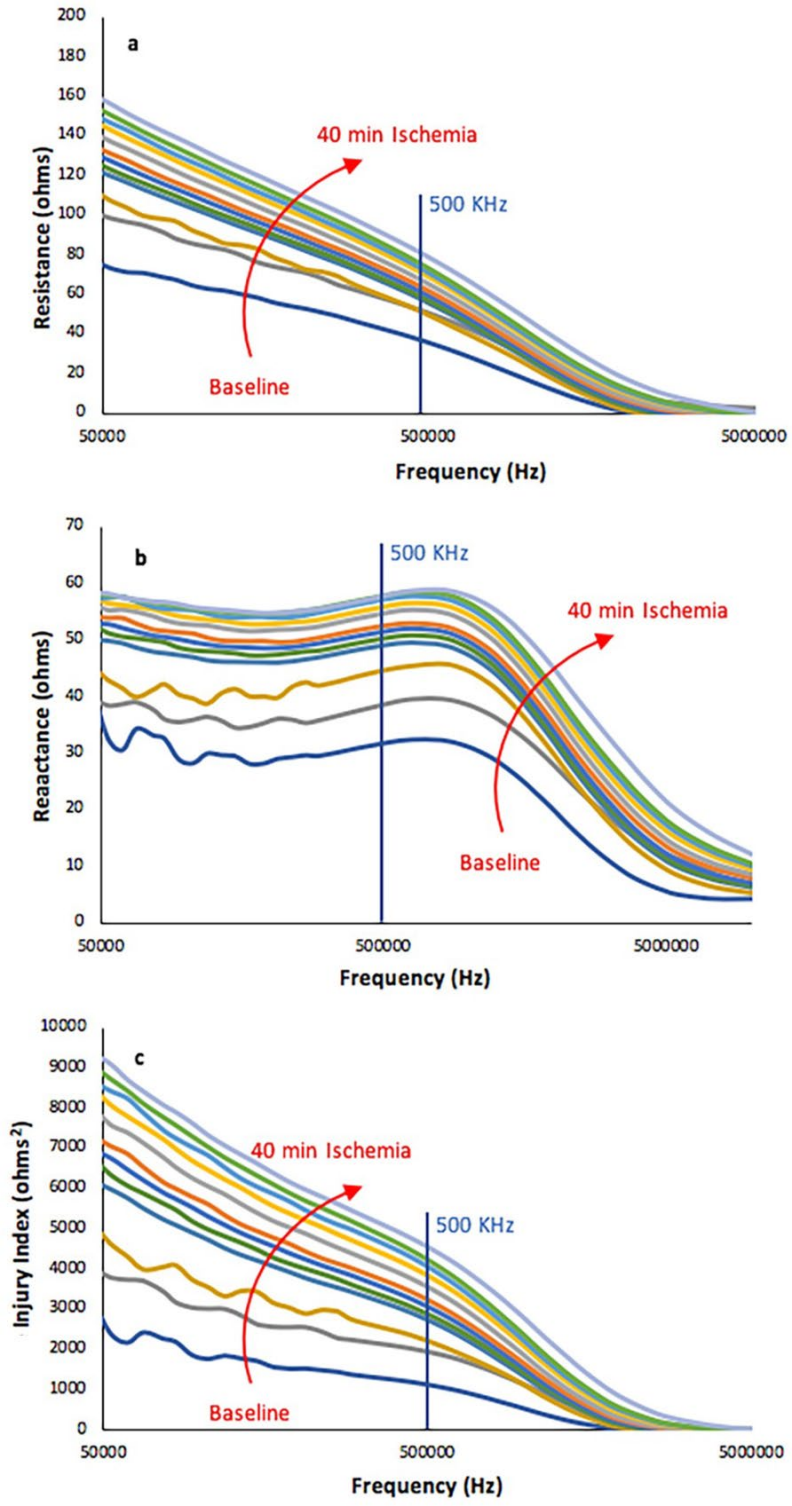
Resistance (**a**), Reactance (**b**), and Injury Index (**c**) change over time with progressive ischemia in a representative compartment syndrome rat model. Both impedance features and the injury index indicate consistent multifrequency changes in rat muscle. Time stamps for graphs: Baseline and Inflated tourniquet (bottom two lines), followed by ischemia times from 2 to 40 min.

We observed alterations in the multi-frequency EIM data, including elevations in both resistance and reactance values shown in Fig. 4a,b, respectively (data from a representative animal). The most dramatic changes occurred within just minutes of initiating the blood pressure cuff inflation and continued to develop for over 40 min. Changes were present across the frequency spectrum. Critically, these elevations in both impedance parameters were consistent with previous cardiac work cited above and predicted by impedance theory^17,18,24–26^.

**Figure 5:**
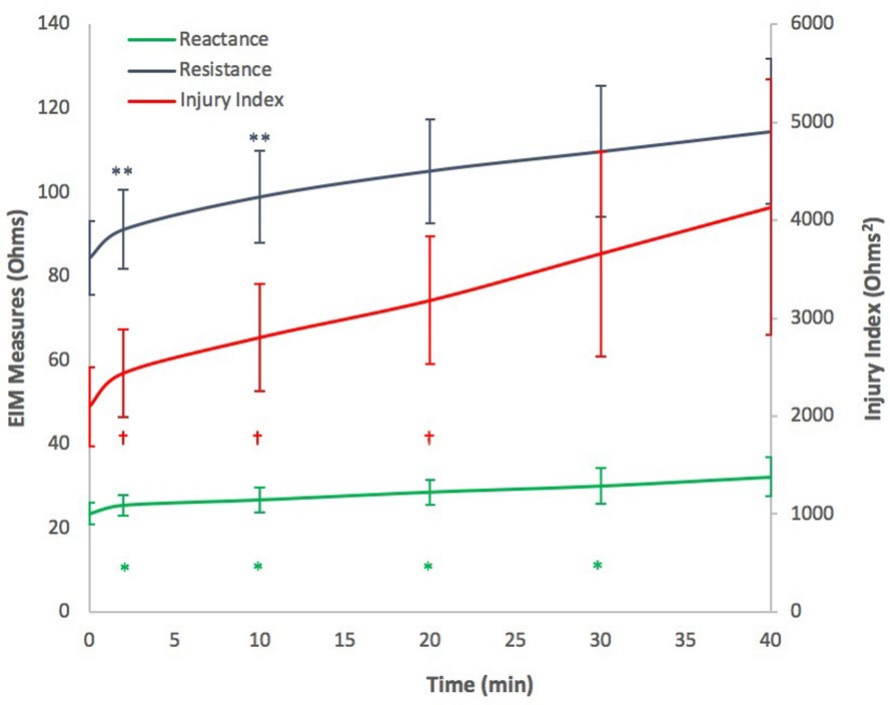
Average reactance, resistance, and injury index (± standard error of mean) values for 12 rats at 450 kHz. *Significant reactance values compared to 0 (baseline tourniquet inflation), 0.046, 0.046, 0.03 and 0.02. **Significant resistance values compared to 0 (baseline tourniquet inflation), 0.031, and 0.032. ^†^Significant injury index values compared to 0 (baseline tourniquet inflation), 0.007, 0.032, and 0.048.

Figure 5 and Table 1 present the 450 kHz reactance, resistance, and injury index values averaged across all animals over the 40-min ischemia injury period (measurements for 2, 10, 20, 30, and 40 min are shown here only). Time 0 indicates the first measurement upon inflation of the tourniquet. Reactance, resistance, and injury index significantly changed over time (p = 0.047, p = 0.0005, and p = 0.024, respectively), with time-specific p values reported in Fig. 5.

**Table 1:** Resistance, Reactance, and Injury Index values at 450 kHz from baseline onward (no cuff pressure).

Time (min)	Reactance (X, Ω)	Resistance (R, Ω)	Injury Index (Ω^2^)
0	23.45 ± 6.95	84.27 ± 23.20	2,095.49 ± 406.29
2	25.51 ± 6.59	91.13 ± 25.04	2,438.18 ± 447.33
10	26.78 ± 7.85	98.92 ± 29.00	2,803.19 ± 545.86
20	28.61 ± 7.92	105.10 ± 32.67	3,182.36 ± 653.68
30	30.04 ± 11.37	109.73 ± 41.28	3,659.26 ± 1,045.64
40	32.14 ± 12.30	114.48 ± 45.71	4,133.76 ± 1,307.92

**Figure 6:**
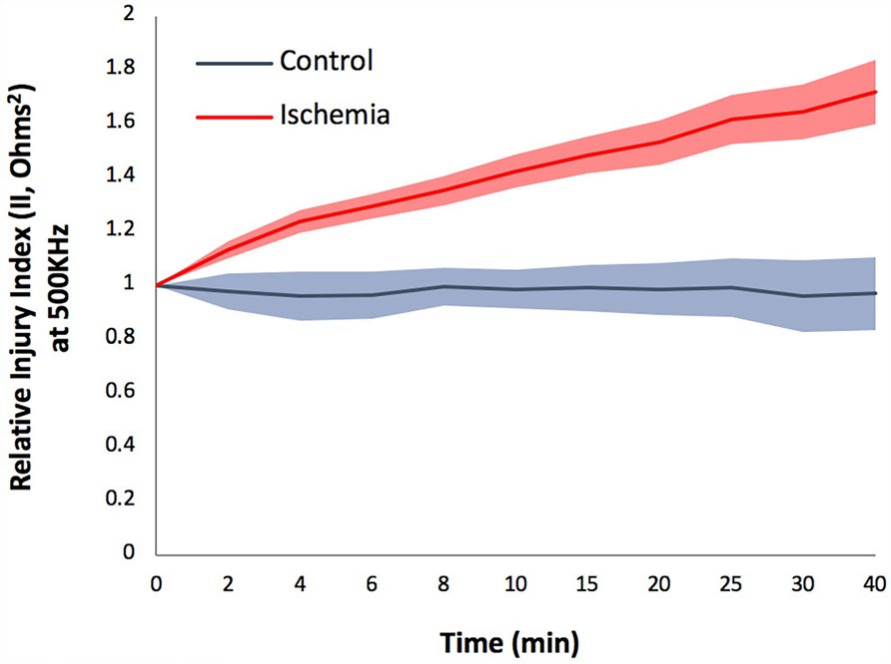
Relative injury index (RII) at 450 kHz over time in a group of animals (n = 12) developing ischemia (red) and a group of non-injury control animals (n = 5) in which the blood pressure cuff was inflated to only 30 mmHg and no tourniquet was placed (blue). Shadow areas represent ± standard error of the mean.

Figure 6 shows the relative injury index (RII) over time for ischemia (n = 12) and control (n = 5) groups, demonstrating a significant difference between the control and ischemia groups for all time points from 2 min onward (p = 0.0001). This further indicates that the observed changes are directly related to the induced injury in the limb.

## Discussion

The diagnosis of compartment syndrome remains a challenge. Overlooking it entirely or diagnosing it late has major negative clinical implications. It is often referred to as a “clinical diagnosis,” in that physicians must balance various factors, including pain, physical exam findings, and subjective estimations or objective measurements of compartment pressure to determine whether compartment syndrome is present or impending^3,6^. Yet the crucial physiological features—inadequate tissue perfusion and associated ischemia—are only indirectly evaluated by these approaches, including direct compartment pressures. In this study, we hypothesized that EIM could provide a better indicator of muscle hypoperfusion.

The experimental model used to test our hypothesis appeared to generate reproducible reduced perfusion with hypoxia, as confirmed by the Hypoxyprobe staining of both vascular endothelial cells (intense red staining) and myocytes (diffuse red staining) within the muscle from the limb undergoing study; no such positive staining was evident in the muscle excised from the contralateral uninjured limb. We observed a significant escalation in both EIM resistance and reactance values during the 40 min of ischemia. This increase in resistance and reactance was absent when measurements were collected from healthy animals not subjected to ischemic injury. As such, the EIM signal showed reproducibility and stability under normal circumstances.

Additional explanations of the EIM-associated alterations in this model are required. In EIM, a weak current is passed between electrodes, and the resultant voltage measurements reflect the muscle condition through which this current passes. Several tissue features contribute to the measured impedance in a complex fashion^27^. Among these include the amount of free intracellular and extracellular water, membrane integrity, ion concentrations, and capillary blood flow. Reduced tissue perfusion would be expected to have many effects, including reducing free water, inhibiting blood flow, and causing membrane instability. Cumulatively, these have the impact of increasing the measured reactance and resistance values. Moreover, ischemia directly affects cellular structures, in this case causing swelling of the cells (i.e., increased intracellular space) with a concomitant reduction in extracellular space, two alterations that would also be expected to increase both resistance and reactance values across the frequency range.^18^ Thus, the impedance-based alterations observed here reflect measures of tissue biomolecular properties related to reduced perfusion and ischemia than they do the clinical measures and compartmental pressures currently utilized.

While resistance and reactance values both showed similar changes, combining the two measures and creating the RII, the relative ischemia injury index, provided an even more consistent measure of ischemic change over time, as shown in the multifrequency plot in Fig. 4c. Since both resistance and reactance measure different aspects of tissue health, this approach helps to ensure the greatest sensitivity to ischemic change.

Although multifrequency data are interesting to demonstrate in graphical form, it will be helpful to focus on just a single frequency or a limited set of frequencies to develop a simpler monitoring mechanism. To that end, we compared several different individual frequencies; while many would be usable, the frequency closest to 450 kHz (precisely 446,683 Hz) appeared very reliable across individual animals (data not shown) and thus was used in additional analyses.

It is important to note that ischemia from clinical ACS occurs on a continuum, and the degree of permanent muscle damage is related to the time course of exposure to hypoxia. Early in its time course, muscle changes from ACS may be partially or wholly reversible, while prolonged exposure can be sufficiently severe to lead to irreversible damage. The resistance and reactance data both demonstrate increasing alteration over time; combining both measures into a single index, the RII, we believe, will provide greater sensitivity to early injury. With increasing exposure to hypoxia, the RII increases, demonstrating the most rapid increases during the earliest time points. These differences are particularly obvious in comparison to the controls. Since these measurements can be made noninvasively, the contralateral limb could be used as a simple internal control in a patient suspected of potentially developing compartment syndrome. Further, with this approach, control measurements are unlikely to be influenced by the mean arterial pressure in the same manner that compartment pressures can be affected, as the measurements are a more direct means of assessing tissue hypoperfusion and resultant hypoxia.

While our data presented here suggest valuable potential for using EIM to facilitate the early detection of ACS, these findings must be taken in context, and translating from a rat model to a clinical setting may have challenges. For example, our measurements were performed transcutaneously. Rat skin and subcutaneous tissues are considerably thinner than humans, and exactly how well this technique will translate is unknown. But the fact that the skin is relatively thin also means that any skin injury induced by the procedure itself is unlikely to substantially impact the overall bulk impedance measured here. Nevertheless, it is certainly possible that alterations to its properties due to sweating, subtle injury, and intradermal blood flow could all impact the data to some extent. EIM has indeed been used transcutaneously for quantifying muscle health in humans, but whether these findings will carry over to the setting of ACS is also uncertain. Furthermore, compartment syndrome can be insidious in its effects, and it is not uncommon to find some muscles within a given compartment being heavily affected by ACS while adjacent muscles are spared permanent damage^28,29^. Whether this heterogeneity will reduce EIM’s sensitivity to effect is uncertain. This model presents with limitations, as do all animal models. It is impossible to know whether the very high pressure from the blood pressure cuffs could have resulted in some degree of contusion injury along with the ischemic change. This is unlikely since the histological features observed are quite distinct from those of contusion injury. Finally, it remains entirely uncertain whether EIM can sense the earliest stages of developing ACS or whether alterations will only become apparent when the condition has advanced. Nevertheless, the potential for the data to be collected frequently, even in the presence of a cast (via electrodes placed within the cast itself before its placement), could help improve our ability to detect subtle but significant changes indicative of incipient ACS. Full development and clinical testing in large animal models or humans will address these questions more specifically.

It is appropriate to consider these findings as only a preliminary first step, and even if EIM ultimately succeeds as a diagnostic tool for ACS, it could only be used in close association with sound clinical judgment; even if successful, it seems unlikely that EIM could ever be used as the sole tool to diagnose ACS. Yet the data presented here make a compelling argument for additional investigations into using EIM to recognize muscle hypoperfusion and ischemia early. These are the hallmarks of ACS and, ultimately, the underlying mechanism of tissue destruction. The fact that EIM is sensitive to these alterations bodes well for its future application in the prevention of this devastating condition.

## Data Availability

The datasets used and/or analyzed during the current study are available from the corresponding author upon reasonable request.
